# Effect of a Tai Chi-Based Exercise Program on Cognitive Function Among Older Adults With Dementia

**DOI:** 10.1093/geroni/igaa057.604

**Published:** 2020-12-16

**Authors:** Rhayun Song, Myonghwa Park, Misook Jung, Hyun Li Kim, Hanna Lee

**Affiliations:** 1 Chungnam National University, Dae Jeon, Republic of Korea; 2 Chungnam National University, Daejeon, Taejon-jikhalsi, Republic of Korea; 3 Chungnam National University, Daejeon, Republic of Korea

## Abstract

Cognitive function with dementia is strongly associated with physical function decline. A low intensity aerobic exercise, such as Tai Chi, may help either prevent or slow down cognitive impairment. This study aimed to examine the effect of Tai Chi-based exercise program on cognitive function among older adults with early dementia. Individuals registered at the Dementia Support Center were invited to participate in the Tai Chi-based exercise program twice a week for one-hour session for 20 weeks. The comparison group with the same inclusion criteria but not participated in any formal exercise program were recruited by matching with age, education, and pre-cognitive function. Cognitive function (MOCA-K) was measured at the pretest and at the completion of the study period. Fifty-two older adults with dementia (29 in Tai Chi group, 23 comparisons) with the mean age of 80.5 years completed all measurements. All participants had at least one chronic disease. About 50% of the participants received no formal education. At the completion of the study, Tai Chi group improved their cognitive function, while their counterpart remained similar in their MOCA-K score, specifically in attention (F=5.21, p=.027) and short term memory recall (F=6.66, p=.013). In conclusion, Tai Chi-based exercise program was safely and effectively applied to older adults with early dementia. The participants were able to follow the movements with the attendance rates of 80% during the study period. Further studies are warranted to explore the relationship between physical exercise and cognitive function in this population with various types of cognitive impairment.

